# The psychometric properties of a tool to assess brief behaviour change counselling in South Africa

**DOI:** 10.4102/phcfm.v12i1.2540

**Published:** 2020-12-22

**Authors:** Jani Fouche, Robert Mash, Zelra Malan

**Affiliations:** 1 Division of Family Medicine and Primary Care, Faculty of Medicine and Health Sciences, Stellenbosch University, Cape Town, South Africa

**Keywords:** directive counselling, educational assessment, lifestyle risk reduction, primary health care, process health care assessment, reproducibility of results

## Abstract

**Background:**

Primary care providers should be competent in brief behaviour change counselling (BBCC). A new model of BBCC was developed in South Africa. Tools are needed for training and research to evaluate BBCC.

**Aim:**

To evaluate the validity and reliability of a tool to assess BBCC.

**Setting:**

Primary care providers in Western Cape, South Africa.

**Methods:**

Exploratory sequential mixed methods included initial qualitative feedback from an expert panel to assess validity, followed by quantitative analysis of internal consistency, inter- and intra-rater reliability. Six raters assessed 33 randomly selected audiotapes from a repository of 123 tapes of BBCC at baseline and 1 month later.

**Results:**

Changes to the existing tool involved item changes, added items and grammatical as well as layout changes. The ‘Assessment of Brief Behavioural Change Counselling’ tool (ABC tool) had good overall internal consistency (Cronbach’s alpha 0.955), inter-rater (intra-class correlation coefficient [ICC] 0.813 at follow-up) and intra-rater reliability (Pearson’s correlation 0.899 and *p* < 0.001). Sub-scores for the Assist (ICC 0.784) and Arrange (ICC 0.704) stages had lower inter-rater reliability than the sub-scores for Ask (ICC 0.920), Alert (ICC 0.925) and Assess (ICC 0.931) stages.

**Conclusion:**

The ABC tool is sufficiently reliable for the assessment of BBCC. Minor revisions may further improve the reliability of the tool, particularly for the sub-scores measuring Assist and Arrange. The ABC tool can be used in clinical training or research studies to assess fidelity to this model of BBCC.

## Background

Behavioural risk factors, underlying both communicable and non-communicable diseases (NCDs), have been clearly described in the South African (SA) context.^1^ These include unsafe sex, tobacco smoking, alcohol use, interpersonal violence, physical inactivity and unhealthy diet.^1^ The Department of Health has identified the need for health professionals to have capability in behaviour change counselling as one of the strategies to address these factors, particularly in relation to NCDs such as diabetes and hypertension.^2^ Behaviour change counselling by primary care providers has the potential to reach more people and to target those at risk as well as those with established diseases.^2^ The World Health Organization recommends brief advice or counselling as an effective intervention for tobacco cessation, harmful alcohol use, physical inactivity and unhealthy diets.^3^

Health services in the public sector of SA serve approximately 80% of the population, and primary care providers work under significant time pressure because of the high workload.^4^ Behaviour change counselling by primary care providers must therefore be brief in order to be a feasible strategy. Primary care is nurse-led with support from doctors, and therefore nurse practitioners have the greatest opportunity to provide such counselling. In some settings, counselling is also offered by non-professional health workers such as lay counsellors and health promoters.

Current health workers lack knowledge of lifestyle modification and express a number of barriers to brief behaviour change counselling (BBCC).^5^ Barriers include poor compliance of patients to health workers’ advice and consequent demotivation of health workers, lack of time, language barriers and poor counselling skills.^5^ The pre-service training of primary care nurses and doctors has inadequate training in BBCC.^6^

In an attempt to address the training of primary care providers, SA researchers developed a new model of BBCC.^7^ This model integrated the well-known 5 As (Ask, Advise, Assess, Assist and Arrange) approach with a guiding style derived from motivational interviewing (MI).^7^ Although MI includes a less structured approach, as well as specific strategies and communication skills; at its core is the ‘spirit of motivational interviewing’ and what is referred to as a guiding style.^8^ The guiding style is characterised as a collaborative, evocative, empathic, respectful and focussed approach to the person. The evocation of the patient’s own desire, ability, reasons, need or commitment to change behaviour is the active ingredient in making MI work. The guiding style is key to helping people change behaviour and is congruent with a person-centred approach.^9^ It is preferred to the more authoritarian and directive style, often adopted by health professionals^9^ and embedded in the original descriptions of the 5 As approach.^10^

There is good quality evidence for the effectiveness of MI in reducing alcohol consumption, weight and blood pressure.^10^ There is also limited evidence for the effectiveness of the 5 As approach in reducing tobacco smoking, alcohol and weight.^10^ The 5 As approach has been endorsed by a number of professional bodies in Australia, Canada and the United Kingdom.^11,12,13,14^ The new BBCC model was shown to be effective in a quasi-experimental study of smoking cessation in pregnant women in Cape Town.^15^ This study found that an additional 5% – 8% of women stopped tobacco smoking and 12% – 13% of women reduced tobacco smoking in a public sector antenatal clinic when compared with usual care.

A training programme was developed for primary care providers and shown to be effective in changing clinical practice.^16,17^ The study showed that nurse practitioners and doctors were significantly better at a guiding style and completing the 5A steps after training and that this persisted into clinical practice 6 weeks later.^17^ Primary care providers were trained to incorporate BBCC into their consultations when behaviour change was a particular focus. They were trained over 8 h in the 5A steps, the guiding style, specific communication skills such as the use of open questions and information exchange as well as knowledge of lifestyle modification goals and strategies.

A draft tool was also developed to assess the ability of primary care providers to offer BBCC in consultations. However, the validity and reliability of this assessment tool needed further evaluation. No existing assessment tools for MI, the 5 As or BBCC adequately evaluated this new model of BBCC.^18,19,20^ Such a tool would be valuable to future research studies that further investigate the effect of the BBCC model in our context, as well as to clinical trainers who want to observe and provide feedback to students or primary care providers on their practice of BBCC.

The aim of this study, therefore, was to evaluate the psychometric properties of a tool to assess this new model of BBCC. Specific objectives were to determine the content validity, internal consistency and reliability of the tool.

## Methods

### Study design

This was an exploratory sequential mixed methods validation study. Initial qualitative feedback on the content validity of the tool by an expert panel informed the revision of the tool, which was then quantitatively analysed for internal consistency and reliability.

### Model of brief behaviour change counselling

Table 1 outlines the key tasks to be accomplished at each of the five steps as per the model of BBCC. The guiding style was characterised as:

- Collaborative – encourages interaction and collaboration.
- Evocative – elicits the patient’s viewpoint, experience and solutions.
- Empathic – demonstrates active and accurate listening.
- Respectful – accepts the patient’s choices and control over their own life.
- Focussed – focusses on a specific behaviour and series of steps.

**TABLE 1:** Comparison of items between the original and revised tool after feedback from a three member South African expert panel in 2018.

Section of tool	Original items	Revised items	Reasons for change
Ask	Asks about the risk behaviour. Asks what the patient already knows or would like to know. Asks permission to discuss the issue.	1. Asks if the risk behaviour is present. 2. Asks about the risk behaviour. 3. Asks what the patient already knows or wants to know about the risk behaviour. 4. Asks permission to provide further information.	Items needed to differentiate between asking if a risk behaviour was present and asking about the extent and nature of that behaviour.
Alert	Provides information tailor made to the patient’s need. Provides information in a neutral way. Elicits the patient’s response to the information provided.	5. Provides information related to what the patient already knows or wants to know about the risk behaviour. 6. Provides additional information in a neutral way. 7. Asks for the patient’s response to the information provided.	Item 5 needed to make the link to the exploration of the patient’s perspective in item 3 more explicit. Item 6 made it clearer that this refers to additional information provided by the practitioner.
Assess	Assesses importance of change for the patient. Assesses the patient’s confidence to change. Confirms the patient’s readiness to change and respect the patient’s autonomy in their decision or choice.	8. Assesses importance of change for the patient. 9. Assesses the patient’s confidence to change. 10. Confirms the patient’s state of readiness. 11. Respects their choice.	Item 10 was phrased more neutrally as the ‘state of readiness’ to not imply that the patient should be ready. Item 11 was retained, but as a separate item.
Assist	Clarifies the goal for change. Discusses different options or strategies available with the patient. Provides relevant practical assistance like leaflets and/or telephone numbers.	Not ready 12. Asks about or acknowledges the patient’s concerns regarding change. 13. Asks the patient to think of realistic ways to overcome these concerns. 14. Offers supportive material. Ready 15. Clarifies the specific goal for change. 16. Agrees on what action the patient will take. 17. Offers relevant, practical assistance, e.g. supportive material, contact details for community-based resource services. 18. Helps the patient identify social support for change.	Items needed to be separated, depending on whether they applied to a person who was ready or not ready to change. The original items implied the patient should be ready to change. Additional and more specific items were added from the training manual.
Arrange	Arranges for follow-up appointment. Displays empathy by demonstrating moral support. Involves the patient’s social support in the follow-up (friends and/or family).	Not ready 19. Emphasises that help is available when ready. Ready 20. Arranges a follow-up contact to provide on-going support and review progress. 21. Refers for expert or additional help if appropriate. 22. Emphasise your on-going commitment to support change.	Again, items were separated into those that applied to patients who were ready or not ready to change. Identifying social support was seen as part of ‘assist’. Displaying empathy was seen as part of the guiding style and not a specific item for this step.

### Content validity

An expert panel was used to assess whether all the items in the draft tool should be included, whether any important items were missing and that each item was phrased in such a way that it accurately described the criterion to be observed.^21^ The expert panel consisted of the three researchers who developed the model of BBCC, two of whom were academic family physicians and one a social scientist. All three were also recognised as members of the international Motivational Interviewing Network of Trainers.

The original assessment tool (Table 1) mirrored the five steps and assessed the extent to which the practitioner completed the tasks for each step. If they performed none of the tasks at the specific step, they scored 0; if they performed one or two tasks, they scored 1; and if they completed all three tasks, they scored 2.

Qualitative feedback on the content of the tool was obtained via a 4-h group discussion with the expert panel. The content of the original tool was juxtaposed with the model of BBCC as described in the ‘Helping people change’ training manual.^22^ The manual was written to train practitioners in BBCC. The experts were asked to comment on the tool’s content and whether any tasks or competencies needed to be excluded or added. The construction of the tool and how the questions were phrased were also discussed to ensure conceptual clarity and alignment with the tasks being evaluated. Finally, the layout and appearance of the tool were discussed. Comments were clarified, consensus reached and the discussion was recorded on audiotape.

The researcher analysed the content of the audiotape recording and her own notes to extract the key feedback on the content, construction and appearance of the tool. A new version of the tool was formulated. Respondent validation with all three experts was conducted via email over four iterative rounds until the panel was all satisfied with the final tool.

### Internal consistency

A previous study^17^ recorded 123 consultations from 41 primary care providers (23 nurse practitioners, 12 family medicine registrars, 2 general practitioners and 4 family physicians) with standardised patients over a 6-week period. A standardised patient is an actor who has been trained to simulate a patient in a consistent role play. The audio recordings were made during a BBCC training course that involved all the practitioners. Recordings were made before, immediately after and then 6 weeks after BBCC training.

To measure internal consistency, a selection of recordings was made with the help of the Biostatistics Unit at the Faculty of Medicine and Health Sciences, Stellenbosch University. Assuming 90% power, a difference in the mean total score of 0.1, a standard deviation of 0.2 and a significance level of 0.05, the sample size required to determine internal consistency was 33. Thirty-three audiotapes were randomly selected from the pool of 123 audiotapes.

Each audiotape was scored by six independent raters by using the tool. The raters included the primary researcher, three experts in BBCC from the original study, a final-year family medicine registrar and a primary care nurse practitioner trained in BBCC. The primary researcher, the final-year family medicine registrar and the nurse practitioner work in the clinical setting, and all three attended training in BBCC. The three expert raters were from the academic setting and were part of the development of the initial tool as well as the training course in BBCC. No specific training was conducted on the use of the tool. All six participants had to listen to 33 audiotapes of a BBCC session between a health care worker (either a nurse or a doctor) and a simulated standardised patient who wanted to discuss one of the four risk factors for NCDs (tobacco smoking, alcohol use, physical inactivity or unhealthy diet).

There were 22 different items in the revised tool that required assessment. Each item was assessed with a tick if the practitioner did the task, a cross if the task was not performed or as not applicable if the task was deemed not relevant. For steps 4 (Assist) and 5 (Arrange), different sections of the tool needed to be completed, depending on whether the patient was ready or not ready to change. The rater had to decide which section to complete and only complete the appropriate section. These separate sections did not have the same number of items, and therefore the denominator for the total score differed between those who were ready to change (denominator of 18) and those who were not ready to change (denominator of 15). If the rater completed both sections, then all items were included in the calculation of the total score (denominator of 22). If items were marked as not applicable, then the denominator was decreased to only include the number of applicable items. Items were scored as 1 if the task was completed and 0 if the task was not completed, to calculate a total score for the assessment. A percentage score was calculated for each assessment based on the total score as the numerator and the appropriate denominator.

Data were captured in Excel, checked for errors and imported to the Statistical Package for Social Sciences version 25 (SPSS) for analysis. Internal consistency was analysed by using Cronbach’s alpha for the total percentage score and the sub-scores for each of the five steps. This equated to a number between 0 and 1, where 0.70 and above was seen as a good internal consistency.^23^

### Reliability testing

For reliability testing, the same sample of 33 audiotapes was used. In addition to the initial assessments of each tape as explained above, the 33 audiotapes were re-assessed 1 month later by the same raters.

All data were again captured on an Excel spreadsheet and entered into SPSS for analysis. An intra-class correlation coefficient (ICC) was calculated to test for inter-rater reliability for the total percentage score, sub-scores and for each item in the tool. An acceptable ICC was considered to be 0.70 or higher.^23^

Pearson’s correlation was used to calculate the correlation between the assessment of the audiotapes at baseline and then 1 month later. Good correlation was shown by a coefficient greater than 0.7 and a *p* < 0.05.

### Assessment of competency

The global assessment of competency was a score, which each rater added at the end of their assessment of each audiotape (1 = not competent, 2 = borderline or unsure, 3 = competent and 4 = excellent), based on their expert judgement of overall performance. Borderline regression, which is a recognised method for creating a standard in assessment, was used to regress the total percentage score against the global assessment to obtain a pass score for the competent practitioner.

## Ethical consideration

The data used for this study were previously ethically approved by the Health Research Ethics Committee at Stellenbosch University, for a doctoral study, on 12 February 2012 (N11/11/321). This further study was ethically approved by the same committee on 24 April 2018 (S17/11/271).

## Results

### Content validity

Table 1 shows the comparison of the items in the original tool with the items in the revised tool as a result of the feedback from the expert panel. It also provides some feedback on the reasons for changing items. Overall, 10 items were added to the tool, 1 item was deleted from the tool and 8 items were rephrased. Minor grammatical errors were corrected. The placement of four items was changed, and the sections for Assist and Arrange were divided into different items depending on whether the client was ‘ready to change’ or ‘not ready to change’.

The scoring system was changed from an assessment of each section of the tool to an assessment for each individual item as yes (score 1), no (score 0) or non-applicable (no score).

A number of additional supporting sections were added to the tool:

- Background information: Name of the assessor, practitioner and the date of assessment.
- Scoring information: Instructions on how to score and space to record the score.
- Feedback: A space for written formative feedback to the practitioner.
- Anchors: Notes to define each item and to standardise observations to assess if the item was performed or not.

Finally, the panel suggested naming the tool the ‘Assessment of Brief Behavioural Change Counselling’ (the ABC tool).

### Internal consistency

**FIGURE 1:**
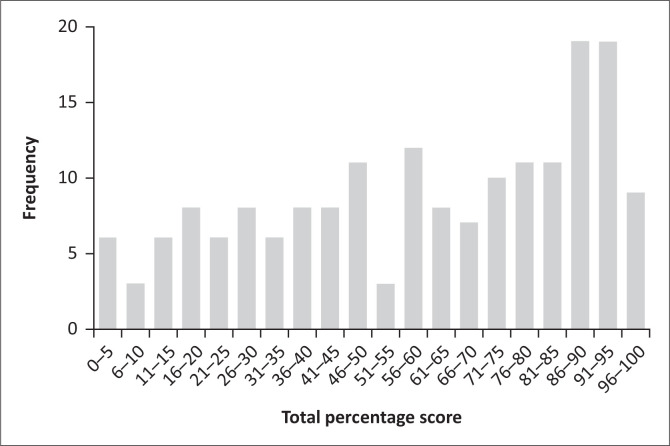
Distribution of raters’ scores (*N* = 197).

The distribution of total percentage scores for all six raters and 33 tapes is shown in Figure 1, and the median score was 65% (interquartile range [IQR]: 38.5–87.0). One rater failed to score one tape, making the total number of scored tapes 197.

Cronbach’s alpha for the total score was 0.955, suggesting good internal consistency. Cronbach’s alpha for each of the 5 As in set 1 (first rating of tapes) and set 2 (second rating of tapes) is shown in Table 2. Cronbach’s alpha for each of the steps was greater than 0.700 suggesting good internal consistency and consistently greater than 0.900 for steps 1 to 3.

**TABLE 2:** Internal consistency of the ABC tool for 33 audiotapes assessed by six independent South African raters in 2018.

Variable	Set 1 Cronbach’s alpha	Set 1 ICC	Set 2 Cronbach’s alpha	Set 2 ICC
1. Ask	0.928	0.920	0.925	0.901
2. Alert	0.934	0.925	0.939	0.926
3. Assess	0.939	0.931	0.942	0.931
4. Assist	0.858	0.784	0.906	0.834
5. Arrange	0.778	0.704	0.782	0.742

ICC, Intra-class correlation coefficient.

### Reliability testing

The ICC for the total score in sets 1 and 2 was 0.714 and 0.813, respectively. Table 2 shows the ICC for each of the 5A steps and Table 3 shows the Cronbach’s alpha and ICC for individual items. The ICC for steps 4 and 5 was much lower than the other three steps, although all were above 0.7 suggesting acceptable inter-rater reliability for the sub-scores. The ICC for each of the items was less than 0.7 for all items apart from item 9. This suggested poor inter-rater reliability at the level of individual items.

**TABLE 3:** Internal consistency and inter-rater reliability of individual items in the ABC tool for 33 audiotapes assessed by six independent South African raters in 2018.

Item number	Cronbach’s alpha	ICC	Item number	Cronbach’s alpha	ICC
1	0.825	0.311	12	0.818	0.409
2	0.613	0.197	13	0.612	0.196
3	0.908	0.609	14	0.802	0.402
4	0.928	0.673	15	0.801	0.354
5	0.826	0.431	16	0.727	0.302
6	0.883	0.532	17	0.870	0.529
7	0.897	0.566	18	0.844	0.479
8	0.899	0.579	19	0.819	0.418
9	0.959	0.800	20	0.881	0.527
10	0.776	0.326	21	0.851	0.486
11	0.740	0.296	22	0.768	0.339

ICC, Intra-class correlation coefficient.

Test–retest reliability was assessed by using Pearson’s correlation (Table 4). Steps 1 to 3 as well as the total score showed good test–retest reliability, whilst steps 4 and 5 had a correlation less than 0.7.

**TABLE 4:** Intra-rater reliability of six South African raters at baseline and 1 month later for 33 audiotapes by using the ABC tool in 2018.

Step	Pearson’s correlation	*p*
1. Ask	0.824	< 0.001
2. Alert	0.860	< 0.001
3. Assess	0.823	< 0.001
4. Assist	0.619	< 0.001
5. Arrange	0.478	< 0.001
**Total Score**	**0.899**	**< 0.001**

### Assessment of competency

Borderline regression of the global scores against the total percentage scores gave a cut-off score of 54.8% for the minimally competent practitioner. Using this cut-off score, 60.9% of practitioners were judged as competent.

## Discussion

The ABC tool’s total score had good internal consistency, inter- and intra-rater reliability. The sub-scores for all of the 5A steps also had good internal consistency and inter-rater reliability, although step 4 (Assist) and step 5 (Arrange) had insufficient intra-rater reliability. The overall reliability of steps 4 and 5 was less than that of steps 1 to 3. The reliability of individual items was not established.

The lower reliability for the sub-scores in step 4 (Assist) and step 5 (Arrange) may have been because of the division of the tool into scoring for those ‘ready to change’ versus those ‘not ready to change’. Some raters marked both the ready and not ready to change sections, suggesting confusion in how to use the tool for these steps. In some tapes, it was difficult to determine the readiness to change if the practitioner did the ‘Assess’ step poorly. In other tapes, the readiness to change shifted after the initial assessment making it difficult to classify. Poor intra-rater reliability for these steps may also have been because of learning in the group of raters and clarification on how to use the tool for these steps between set 1 and set 2.

Although the sub-scores behaved reliably, there was variation in the way individual items were rated between raters. Therefore, the tool should not be scored at the level of individual items. Items 2 and 13 were particularly poor. It is possible that raters had difficulty distinguishing item 1 (asking if the risk factors was present) from item 2 (asking about the severity of the risk factor). Item 13 (asks the patient to think of realistic ways to overcome these concerns) could conceptually be relevant to counselling those who are ‘ready to change’ and ‘not ready to change’ as even those who are ready to change might need to think about ways of overcoming their concerns or challenges. Raters, therefore, may have found it difficult to only utilise this item for those who are not ready to change. Again, this implies that the instructions were not explicit enough to make a definite choice between scoring items in the ready and not ready to change options. It is also possible that raters did not follow the anchors consistently enough.

To respond to the issues raised and discussed here, the final tool was revised (Online Appendix 1). Item 2 was rephrased to make the distinction from item 1 even clearer. The layout of the tool was revised to improve use of steps 4 (Assist) and 5 (Arrange). Rather than separating items into the two categories of ‘ready to change’ and ‘not ready to change’, the items were combined into one list and the rater given the opportunity to exclude items that were ‘not applicable’ to the person’s readiness to change. This also simplified the scoring of the tool. Future research can assess whether this revised tool has even better internal consistency and reliability.

The level of internal consistency and reliability of the ABC tool was better than comparable tools, such as the combined behavioural change counselling assessment instrument (CBCAI).^19^ The CBCAI had an internal consistency of 0.70 versus the ABC tool of 0.955. Inter-rater reliability for CBCAI was 0.82 versus 0.90 for the ABC tool. Although superficially the tools appear to have similar intentions, the CBCAI was focussed on tobacco smoking, was not structured according to the 5 As, tried to incorporate the stages of change model^24^ and required a higher level of competency in MI strategies and skills. In the SA context, where primary care is usually offered by nurse practitioners,^25^ we wanted a model of BBCC that was simple, structured, could be applied easily to multiple behaviours and embedded the principles of a guiding style, without the need to master more advanced MI.

The Behaviour Change Counselling Index (BECCI)^20^ is a tool that was intended to be briefer to use than the original research instruments such as the Motivational Interviewing Skill Code (MISC) or Motivational Interviewing Treatment Integrity (MITI).^18^ The BECCI focussed on the spirit and principles of MI and did not include the 5 As. The ABC tool had better internal consistency than BECCI (rated 0.71 and 0.63) and similar inter-rater reliability (BECCI was rated 0.90 and 0.79).

It has been noted that many interventional and educational studies do not report on the validity and reliability of the instruments used to measure complex interventions such as behaviour change counselling.^26^ Having validated the ABC tool in this study, it can now be used to improve the quality of future research in our context. Although not formally measured, the raters reported that the tool was acceptable, practical and quick to use with minimal training.

The expert panel that validated the tool consisted of clinicians from the academic setting who are not much active in clinical practice. Input from non-academic practitioners in the clinical setting could have been valuable to identify specific workplace challenges when validating the tool. The raters who were not involved in the validation process had no training in using the tool before the audiotapes were assessed, other than the written instructions on the tool itself. One rater did not enter any data for the global rating of recordings, and therefore the power of the borderline regression was reduced through the loss of these data points. Two audiotapes mixed Afrikaans and English, which two of the raters struggled to understand. To compensate for this, the tapes were translated verbatim by the principal researcher and distributed to all raters. One rater left out data for audiotapes 13 and 17 in set 2 altogether.

Implications include:

- Pre-service training of doctors and nurses as well as postgraduate training of family physicians is beginning to give more attention to our model of BBCC in SA. Departments of Family Medicine at all universities now have the ability to train this model of BBCC. The ABC tool will be useful in the formative and summative assessment of students. For registrars in family medicine, it can also be included in workplace-based assessment and their portfolio of learning.^27^
- In-service training of primary care providers is also giving more attention to upskilling providers in BBCC. In the Western Cape, the Department of Health has included the model in the primary care clinical guideline^28^ and has offered training to providers. The ABC tool will be useful to assess the success of training.
- Research continues on how to integrate BBCC into comprehensive patient education and counselling in SA primary care and to assess its effectiveness in our context. The ABC tool will be useful in such research to assess fidelity to the intervention.

## Conclusion

These results suggest that the ABC tool is sufficiently reliable for the assessment of this new model of BBCC in clinical settings or research studies. Minor revisions may further improve the reliability of the tool, particularly for the sub-scores measuring Assist and Arrange.

## Appendix 1

### ABC tool

**Practitioner ……………………………    Date ……………………………    Assessor ……………………………**

**Table:**

STEP	CRITERIA	√ = done X = not done NA = not applicable
**ASK** Ask about risk behaviour	Asks if the risk behaviour is present (i.e. Do you smoke)	
	Asks about the risk behaviour (i.e. How much do you smoke)	
	Asks what the patient already knows / wants to know about the risk behaviour.	
	Asks permission to provide further information.	
**ALERT** Alert to risks of behaviour / benefits of change	Provides information related to what the patient already knows / wants to know about the risk behaviour.	
	Provides additional information in a neutral way.	
	Asks for the patient’s response to the information provided.	
**ASSESS** Assess readiness to change	Assesses importance of change for the patient.	
	Assesses the patient’s confidence to change.	
	Confirms the patient’s state of readiness.	
	Respects their choice.	
**ASSIST** Provide practical assistance. Remember to mark as not applicable items that are not relevant to the person’s readiness to change	Asks about or acknowledges the patient’s concerns or challenges regarding change.	
	Asks the patient to think of realistic ways to overcome these concerns or challenges.	
	Offers relevant, practical assistance e.g. supportive material, prescription.	
	Helps the patient identify social support for change.	
	Clarifies the specific goal for change.	
	Agrees on what action the patient will take.	
**ARRANGE** Arrange appropriate follow up. Remember to mark as not applicable items that are not relevant to the person’s readiness to change	Emphasise that help is available when ready / Emphasise your on-going commitment to support change.	
	Refer for expert or additional help if appropriate.	
	Arrange a follow-up contact to provide ongoing support and review progress.	

### Scoring

Give one point for each tick to create a score. Convert this to a percentage by dividing the score by the total number of possible ticks and multiplying by 100. The total number of possible ticks is 20. This number can be reduced if some items are marked NOT APPLICABLE.

**Table:**

Score	Percentage

### Feedback to practitioner

**Table:**

### Notes on the criteria

**ASK:** It is assumed that only one risk behaviour will be discussed. It may be necessary to ask if the risk behaviour is present (e.g. Do you smoke?), but if this is already known then this question is not applicable. When asking about the risk behaviour an assessment of severity is done (i.e. How many cigarettes do you smoke?). This could also be done by using some of the questions in the manual.

**ALERT:** Information should alert the patient to the risks of their behaviour or benefits of change and be related to what the patient already knows or wants to know. Other information may be given, for example relating their behaviour to known health problems. Being neutral implies that the information is shared, without also advising or telling the patient what they must do.

**ASSESS:** Importance and confidence to change should be assessed or acknowledged by using a scale, open questions, reflective listening statements or summaries. The practitioner should confirm the patient’s readiness to change and demonstrate their willingness to respect the patient’s choice.

**ASSIST:** Clarify the goal in terms of what, when, who and where: What exactly are you going to do? What might be some of the challenges? What could you do to overcome these challenges? When will you start? Who can support you? Where will this happen? An attainable action plan should be brainstormed and agreed on. If the patient is not ready for change, care should be taken to explore their ambivalence or concerns, without any advice or pressure.

**ARRANGE:** If ready to change, organise a future contact via phone, email or clinic visit. Referral may be to expert counselling (e.g. dietician, social worker) or additional help (e.g. community health worker). If the patient is not ready to change, then keep an open door for when they are ready.

Further information: http://www.ichange4health.co.za/healthcare-professionals/
